# Reported willingness to participate in a hypothetical HIV vaccine trial and its translation to actual participation among healthy adults—Experience from Kenya

**DOI:** 10.1371/journal.pone.0206656

**Published:** 2018-11-02

**Authors:** Delvin Kwamboka Nyasani, Gaudensia Nzembi Mutua, Rose Miroyo Sajabi, Jane Wairimu Ng’ang’a, John Ndungu Gachie, Amos Macharia Maina, Laura Lunani Lusike, Aggrey Omu Anzala, Matthew A. Price, Gloria Omosa Manyonyi

**Affiliations:** 1 KAVI-Institute of Clinical Research, University of Nairobi, Nairobi, Kenya; 2 Department of Microbiology, School of Medicine, College of Health Sciences, University of Nairobi, Nairobi, Kenya; 3 International AIDS Vaccine Initiative, New York, United States of America; 4 Department of Epidemiology and Biostatistics, University of California at San Francisco, San Francisco, California, United States of America; University of Toronto, CANADA

## Abstract

**Objective:**

To evaluate initial reported willingness to participate in a hypothetical HIV vaccine clinical trial and actual participation of volunteers in a longitudinal observational study.

**Methods:**

We recruited HIV negative male and female volunteers aged 18–45 years into a longitudinal observational study at KAVI–ICR Kangemi in Kenya, to serve as a pool from which to draw participants into a phase I HIV vaccine clinical trial. A structured questionnaire was used to collect information regarding willingness to join a HIV vaccine clinical trial in the future. Study follow-up visits were every 6 months.

**Results:**

A total of 105 participants were screened and 100 (M46:F54) were enrolled into the observational study. Ninety- four per cent of those enrolled expressed willingness to participate in a future HIV vaccine trial. Altruism and desire to learn the body’s response to the vaccine were the most motivating factors at 40% and 25% respectively. At the onset of a 40-person phase I HIV vaccine trial, 86 observational study participants who had previously expressed willingness to participate were contacted but only 26 (30%) came for information. All 26 consented to participate and after screening for eligibility, 24 were eligible. Of the 24, 15 were enrolled. These numbers were not adequate; hence the vaccine trial employed other recruitment methods to meet the deficit.

**Conclusion:**

Observational “pools” of cohorts may not provide adequate number of participants into vaccine clinical trials even if they report willingness; therefore supplementary recruitment methods such as direct community recruitment, passive approach, and snowballing need to be in place.

## Introduction

While great strides have been made in HIV treatment, to ultimately end HIV globally, we need a safe, effective, and affordable HIV vaccine to prevent HIV infection[1]. The clinical research process to test such vaccines requires human subjects to participate[2]. However, for a myriad of reasons, researchers encounter many challenges in recruiting and retaining adequate participants into the clinical trials [3].

Several strategies of active and passive participant recruitment exist that may help enrol participants into trials. Active recruitment involves targeting specific individuals, groups or residents typically from a defined area and/or recruiting from a known pool of eligible participants, such as a cohort study. Passive recruitment typically involves informing the community where researchers intend to recruit through flyers, public events, media, posters, social networking, internet postings and waiting for participants to call or walk into the research sites [4–7].

A double-blinded placebo controlled phase I/ II preventive HIV vaccine study conducted in Thailand medical centres found that multiple stages of recruitment allowed potential participants an opportunity to re-evaluate their willingness to participate in the clinical study which ensured their high level of commitment. From the initial 217 participants who had responded to the calls, they ended up with 54 (25%) who enrolled in the study [8]. There was another study conducted in Tanzania which evaluated experiences on recruitment and retention of volunteers in the first HIV vaccine trial phase I/II in Dar es Salaam. They found that stepwise education provision and sensitization recruitment enabled them to recruit sufficient number of participants into the clinical trial. However, it took them a year to recruit 60 participants[9].

In other instances, researchers have conducted hypothetical willingness-to–participate studies among high risk populations[10–13], and a few in general populations [14];[15] to determine participants’ attitudes and perception towards HIV vaccine clinical trials and their readiness to enrol in actual vaccine trials of which majority found high reported trial participation in HIV vaccine trials. However, few have gone to the extent of evaluating if willingness to participate translates to actual participation [16]. Here we present findings from our study, which evaluated translation of a participant’s willingness to participate in a hypothetical HIV vaccine trial into actual enrolment.

## Materials and methods

### Study design

This was a two year longitudinal observational cohort study, implemented in 2009 at KAVI-Institute of Clinical Research (KAVI-ICR). KAVI-ICR consists of two sites, both in Nairobi. One is located at the Kenyatta National Hospital (KNH) and the other at a small clinic in Kangemi. Kangemi is located on the Western part of Nairobi County and is an informal settlement consisting of people of both medium and low socio-economic classes. The study was designed to recruit both adult male and female participants into a cohort to serve as a pool of volunteers, from which participants would be drawn into future studies (observational studies or clinical trials). Participants who rolled over to another study were replaced. While the study commenced in 2009 at the KNH KAVI-ICR site, recruitment at Kangemi KAVI-ICR site commenced in the year 2010. It was aimed at creating a pool of participants for an anticipated phase I HIV vaccine clinical trial that was to start in the year 2011.

### Study subjects

Participants were HIV negative male and female adults aged 18–45 years and willing to give informed consent. Current or past participants in clinical trials were excluded from participation.

### Study procedures

#### Participant recruitment

Peer leaders from the community were identified and trained on the study. These peer leaders then mobilized potential study participants from local communities, institutions, markets, and churches. They informed the potential participants about the study in the field, and then assembled them for initial study information group sessions delivered by KAVI-ICR staff (community mobilizers). Thereafter, the community mobilizers referred interested participants to the Kangemi KAVI-ICR research site for detailed study information seminars. This was followed by individualized informed consent sessions by study nurse counsellors; where participants’ questions or concerns regarding the study were addressed. On average, each participant had 3 individualized sessions before signing the consent to join the study.

#### Consent and screening for eligibility

We administered an assessment-of-understanding (AOU) of consent information to assess participant’s comprehension of the study before signing the study consent. Only two attempts of assessment-of-understanding were permitted. Those who passed this test by correctly answering at least 8/10 questions were offered the study consent form to sign.

Thereafter, we used a structured questionnaire (S1 Appendix) to obtain socio-demographic data, self-reported health status, medical history, HIV risk assessment, and reasons for volunteering to participate in the study. Information regarding willingness to join a future/hypothetical HIV vaccine clinical trial was also collected. Details of what a HIV vaccine clinical trial entailed were not part of the information given in this study. A study nurse counsellor performed pre and post- HIV test counselling then HIV-testing using rapid test kits [17]. Participants who tested HIV positive were not enrolled but referred for further management at a health facility of their choice. About 10mls of blood was drawn from eligible participants for storage.

#### Follow-up procedures

Follow-up visits were scheduled every six months. At every visit, nurse counsellors performed HIV test using rapid test kits after pre and post- test counselling.

#### Recruitment and consent of participants into a recruiting HIV vaccine clinical trial

Starting from February 2011, participants who had expressed willingness to participate in a future HIV vaccine clinical trial were contacted via telephone calls by the study coordinator to come for information regarding a recruiting phase I HIV vaccine trial (IAVI Protocol B003) [18], which required enrolment of 40 participants. Participants who came in were given information about the HIV vaccine clinical trial and their consent to participate was subsequently sought.

### Clinical trial registry name and registration number

The HIV vaccine trial clinical trial was registered on the National Institute of Health U.S. National Library of Medicine ClinicalTrials.gov.

Registry name: Safety and Immunogenicity Study of Ad26-ENVA and Ad35-ENV HIV Vaccines in Healthy HIV-uninfected Adults.

ClinicalTrials.gov identifier: NCT01215149.

### Ethical considerations

The Kenyatta National Hospital-University of Nairobi Ethics and Research Committee (KNH-UON ERC) approved the study, and we obtained written informed consent from each participant. At each study visit, participants were reimbursed for their time and effort.

### Data analysis

SPSS statistics for windows (version 17.0, Chicago, USA) was used for analysis. Descriptive statistics such as frequencies, percentages, mean and standard deviation was used to describe the participant characteristics and occurrences of motivators across various volunteer demographics. Chi-square test was used to compare demographics and sexual risk behaviour of participants who consented to join an HIV vaccine clinical trial with demographics of those who declined. Results are presented in form of text, tables and figures.

## Results

**Fig 1:**
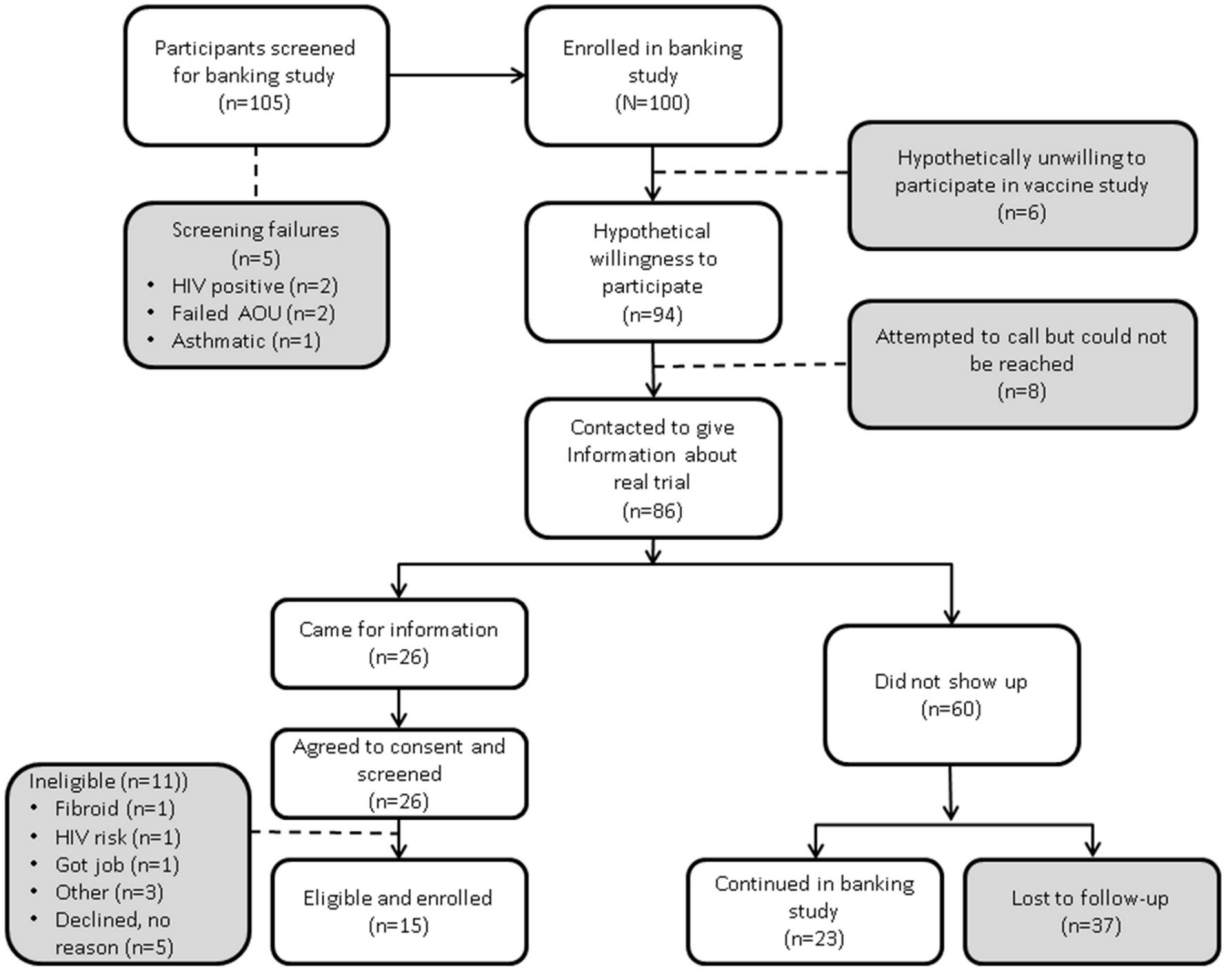
Flow of participant recruitment. These participants were recruited from an observational cohort study into HIV vaccine trial.

A total of 105 participants were screened and 100 (46M: 54F) participants were enrolled into the observational study at Kangemi KAVI-ICR site from September to December 2010 as illustrated in Fig 1.

The majority (77.7%) of the participants were aged 18–29 years, single (56.6%), and students (33.0%) or self-employed (28.0%). Most of the participants reported their perceived general health status as being good or excellent (90.9%) as illustrated in Table 1 below. The main reasons given for motivation to participate in the observational study included altruism (34.0%) chance to know one’s health status (33.0%) and desire to gain knowledge about research (31.0%). While the main motivating factors given for willingness to join a future HIV vaccine clinical trial were:—altruism at 40.4%, desire to learn the body’s response to the vaccine at 25.5%, and misconception about being protected by the HIV trial vaccine at 18.1% (Table 1).

**Table 1:** Characteristics of participants enrolled in observational study and those who expressed willingness to join a hypothetical HIV vaccine clinical trial, and main motivation for participating.

Characteristic	Total enrolled		Total willing	
	(n = 100)		(n = 94)	
	n	(%)	n	(%)
**Mean age = 25, SD = 5.47**				
**Gender**				
Male	46	(46.0)	44	(46.8)
Female	54	(54.0)	50	(53.2)
**Age group**				
18–24	53	(53.0)	48	(51.1)
25–29	24	(24.0)	24	(25.5)
30–34	16	(16.0)	16	(17.0)
35–39	3	(3.0)	3	(3.2)
40–44	3	(3.0)	3	(3.2)
**Marital Status**				
Single	56	(56.0)	52	(55.3)
Married	37	(37.0)	36	(38.3)
Separated	3	(3.0)	3	(3.2)
Divorced	2	(2.0)	2	(2.1)
Widowed	1	(1.0)	1	(1.1)
**Occupation**				
Student	33	(33.0)	29	(30.9)
Self-employed	28	(28.0)	28	(29.8)
Casual-worker	14	(14.0)	14	(14.9)
Housewife	11	(11.0)	10	(10.6)
Professional	7	(7.0)	7	(7.4)
Unemployed	4	(4.0)	4	(4.3)
CSW	1	(1.0)	1	(1.1)
Footballer	1	(1.0)	1	(1.1)
**Self-Reported Health-status**				
Excellent	21	(21.0)	20	(21.3)
Good	69	(69.0)	66	(70.2)
Average	9	(9.0)	8	(8.5)
**Main motivation to participate**				
Altruism	34	(34.0)	38	(40.4)
Desire to learn body response to vaccine	0	(0.0)	24	(25.5)
Presumptive Protection against HIV	1	(1.0)	17	(18.1)
Heroes	0	(0.0)	7	(7.4)
Knowledge	31	(31.0)	5	(5.3)
Knowledge of personal health status	33	(33.0)	3	(3.2)
Access to medical care	1	(1.0)	0	(0.0)

Almost all the participants (94%) expressed willingness to participate in a future/hypothetical HIV vaccine clinical trial, while 6 expressed unwillingness to participate citing fear of side effects, fear of receiving a placebo, indecisiveness and family disapproval as the reasons. Of the 94, 86 were reachable by telephone and invited for clinical trial study information and consent. Twenty-six participants returned and all agreed to join the HIV vaccine clinical trial and were screened (Fig 1). Eleven of the 26 did not enrol: - 2 did not meet the trial inclusion criteria, while 9 were eligible but declined to be enrolled. The reasons for declining enrolment given by 4 of the participants were new job, not ready to use family planning, did not like blood drawn and spouse refusal as indicated in Fig 1. We found no statistically significant differences in social demographic characteristics and the sexual risk behaviour between the 60 participants who had expressed willingness but declined to consent to join the clinical trial and the 26 who consented (Table 2).

**Table 2:** Socio demographic, motivation & sexual risk characteristics of participants in observational study, stratified by consent to participate in HIV vaccine trial.

Characteristic	Total (N = 86)		Consented to actual vaccine trial (n = 26)		Did not consent to trial (n = 60)		P = value
	n	%	n	(%)	n	(%)	
**Gender**							
Male	41	(47.7)	16	(61.5)	25	(41.7)	0.09
Female	45	(52.3)	10	(38.5)	35	(58.3)	
**Age group**							
18–27	58	(67.4)	18	(69.2)	40	(66.7)	0.59
28–37	24	(27.9)	6	(23.1)	18	(30.0)	
38 and above	4	(4.7)	2	(7.7)	2	(3.3)	
**Marital Status**							
Single never married	46	(53.5)	14	(53.8)	32	(53.3)	0.87
Married	35	(40.7)	11	(42.3)	24	(40.0)	
Single ever married	5	(5.8)	1	(3.8)	4	(6.7)	
**Occupation**							
Student	28	(32.6)	7	(26.9)	21	(35.0)	0.58
Self-employed	26	(30.2)	9	(34.6)	17	(28.3)	
Employed	21	(24.4)	8	(30.8)	13	(21.7)	
Unemployed	11	(12.8)	2	(7.7)	9	(15.0)	
**Health-status**							
Excellent	18	(20.9)	6	(23.1)	12	(20.0)	0.93
Good	62	(72.1)	18	(69.2)	44	(73.3)	
Average	6	(7.0)	2	(7.7)	4	(6.7)	
**Main reason for hypothetical willingness to participate**							
Altruism	32	(37.2)	12	(46.2)	20	(33.3)	0.23
Learn response to vaccine	22	(25.6)	3	(11.5)	19	(31.7)	
Presumptive protection against HIV	17	(19.8)	5	(19.2)	12	(20.0)	
Other	15	(17.4)	6	(23.1)	9	(15.0)	
Be a hero	2	(2.3)	2	(7.7)	5	(8.3)	
Interest in research	1	(1.2)	1	(3.8)	3	(5.0)	
Learn their health status	1	(1.2)	1	(3.8)	1	(1.7)	
No response/no answer	2	(2.3)	2	(7.7)	0	(0.0)	
**Sexually active**							0.93
Yes	59	(68.6)	18	(69.2)	41	(68.3)	
No **(duration:)**	27	(31.4)	8	(30.8)	19	(31.7)	0.96
1–5 months	13	(15.1)	4	(15.4)	9	(15.0)	
6–10 months	6	(7.0)	2	(7.7)	4	(6.7)	
11–15 months	4	(4.7)	1	(3.8)	3	(5.0)	
Never had sex	4	(4.7)	1	(3.8)	3	(5.0)	
**Sexual partners in the last one year**							0.57
None	10	(11.6)	2	(7.7)	8	(13.3)	
1	53	(61.6)	16	(61.5)	37	(61.7)	
2	14	(16.3)	4	(15.4)	10	(16.7)	
≥ 3	9	(10.5)	4	(15.4)	5	(8.3)	
**Number of casual partners**							0.64
None	75	(87.2)	23	(88.5)	52	(86.7)	
1	6	(7.0)	1	(3.8)	5	(8.3)	
2	4	(4.7)	2	(7.7)	2	(3.3)	
≥ 3	0		0	(0.0)	1	(1.7)	
**Condom use with casual partners**							0.72
No casual partners	75	(87.2)	23	(88.5)	52	(86.7)	
Never	2	(2.3)	1	(3.8)	1	(1.7)	
Always	9	(10.5)	2	(7.7)	7	(11.7)	
**Injection drug use (IDU)**	0	(0.0)	0	(0.0)	0	(0.0)	
**Sexual partner with IDU**							0.51
No sexual partner	4	(4.7)	1	(3.8)	3	(5.0)	
No	66	(76.7)	22	(84.6)	44	(73.3)	
Don’t know	16	(18.6)	3	(11.5)	13	(21.7)	
**STI in last six months**	0	(0.0)	0	(0.0)	0	(0.0)	
**HIV+ sexual partner**							0.97
Never had sexual partner	4	(4.7)	1	(3.8)	3	(5.0)	
No	79	(91.9)	24	(92.3)	55	(91.7)	
Don’t know	3	(3.5)	1	(3.8)	2	(3.3)	

Those who came in consented for HIV vaccine trial (46% 12/26) had reported altruism as their main reason for wanting to participate. This was not significantly different from the number reporting altruism among those who agreed but did not come in (33%, 20/60, p = 0.26, chi square test). We did note, a significant difference in the percentage of those who reported desire to learn response to vaccine as our main reason, with only 12% (3/26) reporting this among those who came in and consented, compared to 32% (19/60) among those who did not come in (p = 0.05, chi square test).

Of the 60 volunteers who were originally “willing” but ultimately declined to participate, only 23 (38%) continued with their follow-up visits in the observational study. While those who enrolled into the clinical trial were no longer followed in this observational study, their retention in the HIV clinical trial was excellent at 100% (18).

## Discussion

This is the first study from Kenya that has evaluated the utility of an observational cohort study as a potential source of participants for a HIV vaccine clinical trial. Only one in six of our study’s participants who had expressed willingness to participate in a hypothetical HIV vaccine clinical trial ultimately participated in an enrolling trial.

Despite a very high hypothetical willingness to participate, we observed a low actual participation rate. Both the informed consent and the willingness to participate questionnaire used in the observational study did not give detailed information to participants such as: time required and amount of blood to be drawn while participating in a HIV vaccine clinical trial and this may have contributed to the high numbers of actual decliners.

Those who refused hypothetical willingness to participate cited various reasons: fear of side effects, fear of receiving a placebo, indecisiveness and family disapproval based on fear that their family would assume they were infected or could get infected with HIV. However, as we observed very few refusals, we did not see any trends in these data. Others who saw much refusal rates report similar findings, including [19] in which 1516 participants were asked about trial related discrimination and 58% reported negative reactions of friends, family and co-workers following the participant’s self-disclosure of trial participation relating to concerns about the potentially harmful effects of the vaccine on participants health. Also from the same study, participants reported experiencing negative reactions related to assumptions that their participation in HIV vaccine trial meant that they were HIV—infected or at risk of becoming infected with HIV through high risk behaviour.

Health concerns including vaccine safety and side effects, vaccine-induced HIV sero-positivity and the misconception that the vaccine could cause HIV infection have been cited as barriers to participation. This observation however is from a study among an ethnic minority group in the USA [20] and hence may not be generalizable to the African context. Vaccine-induced HIV sero-positivity is a justifiable concern as it may negatively impact an individual’s life including job loss, travel, restrictions on blood and organ donations, and relationship breakups [21]. Researchers should therefore put in place mechanisms to fully inform participants of this possibility and to prevent and mitigate the negative effects of vaccine-induced HIV sero-positivity.

Part of the intention of recruiting participants into a ‘waiting pool’ in an observational study is for study investigators to create trust with participants. However, the participants’ study duration in the observational study (as little as 2 months in some cases) prior to invitation into the HIV clinical trial may not have been adequate to build this trust. This might have also negatively affected their decision to join the recruiting trial, or even to continue with the observational study’s follow-up visits. However, our assumption that participants staying longer in the study could have given us better results might be contrary, as evidenced by a study conducted in the USA among 610 HIV high- risk participants. They found that, as participants stayed longer in the study their hypothetical willingness declined [16].

Our findings are similar to those of a study conducted in the USA, which compared hypothetical and actual willingness to enrol in a preventive HIV vaccine trial among 2531 high-risk HIV uninfected participants from a former Vaccine Preparedness Study (VPS). The study found out that only 20% of those who had stated hypothetical willingness during VPS actually enrolled in the vaccine trial [22]. Another similar study from South Western Uganda evaluated willingness to participate in preventive HIV vaccine trials and possible barriers to participation in a community-based cohort. Of 1013 participants, 95% initially expressed willingness to participate in a future HIV vaccine trial. However, when they were given more information on hypothetical requirements of a HIV vaccine trial such as: the possibility of receiving a placebo or vaccine, large volumes of blood being drawn, frequent visits, and long trial duration; their reported willingness to participate in a future HIV vaccine trial reduced to only 43% [15]. This was also noted among our participants who after receiving information about the HIV vaccine trial, screened and were eligible but declined to enrol.

We found altruism to be an important motivating factor in our observational study even though this did not vary significantly between consenters and decliners into HIV vaccine trial. This had also been cited from a study conducted in the USA. They found that, 80% of their sample of 301 Black Americans had cited altruism as a reason for willingness to participate in AIDS clinical trials [23]. In another study conducted among 2920 Brazilians and Indians, altruism was reported by 55% of participants as a motivating factor[24]. While we did not observe this, another study conducted in Tanzania among 450 young adults found that 50.6% were willing to participate because they had knowledge about vaccine studies [25]

In our study, nearly one out of every five participants’ had a misconception that the vaccine would offer protection against HIV; even though it was not statistically significant this should be a matter of concern for researchers. This misconception was noted as a motivator for study participation especially among those that declined to join the actual trial compared to consenters. This observation implies that a banking protocol may help filter off from trial participation participants with the misconception that trial vaccines provide protection from HIV.

An important aspect of the informed consent process must make clear that the vaccine being tested is experimental and may not provide any protective benefit. Others have observed similar findings including a willingness-to-participate study among Police Officers conducted in Tanzania where the authors reinforce the importance of making clear that an experimental vaccine will not protect against HIV [26]. Also another study conducted in India among men who have sex with men found that potential trial participants overestimated the likely effectiveness of an experimental vaccine, likely leading to decreased condom use [27] Misconception that that the vaccine would offer protection against HIV is a concern to researchers because participants may assume they are “protected” and thus put themselves at increased risk of HIV infection[28].

We also observed a difference in the number who reported an interest in learning the body’s response to vaccine as their main reason for willingness to participate, with significantly fewer among the returnees reporting this compared to those who did not return. Given the modest size of our study, we are not sure we should draw strong conclusions from this observed result, it could simply be that this was not a strong motivating factor among those who actually do return for trial enrollment.

Retention in the observational study of participants who declined to enrol in the vaccine trial was low (about two thirds lost to follow up). Although disappointing, “banking” of participants may perhaps be helpful in filtering out persons who may not complete a clinical trial. It is particularly important that, having received an investigational product, participants in clinical trials adhere to study requirements for safety assessments and for validity of trial results as evidenced by our 100% retention of participants who were recruited into that HIV vaccine trial [18]

### Limitations

Our study did not provide participants with detailed information about what would be required in a HIV vaccine clinical trial. Details such as the amount of blood to be drawn, the numerous visits for safety assessment, and need to avoid getting pregnant were not included. Lack of knowledge about these requirements may have led to the low consent for the clinical trial despite high hypothetical willingness. The observational study’s aim was to follow up the participants for some time to assess their commitment to the requirements of a clinical research study. However, our participants were asked to enrol in the actual vaccine trial after only two months’ stay in the observational study. Perhaps, had they had a longer follow-up period and more detailed information, more participants would have consented for the HIV vaccine clinical trial. Also, we did not have an opportunity to assess the reasons why participants did not respond to our invitation to join the HIV vaccine clinical trial; hence we can say nothing about their motives. Our study had only 100 participants enrolled hence our statistical power was low, since it specifically evaluated participant behaviour with regard to a HIV vaccine trial hence; our findings may not be generalizable to other populations or types of clinical trials. Finally, we note that our data are nearly 10 years old and the epidemic, willingness to participate in clinical research, and motivations for trial participation may have changed. However, because clinical trials still require human subjects to participate, any candid discussion of why volunteers express desire to participate but decline when a trial comes around should help reinforce how difficult it can be to recruit for trials.

Increasing participation in clinical research has become a key area of focus within the National Health Service, with the aim of facilitating evidence–based policy, improving health outcomes and reducing health inequality [29]. Moreover, clinical trials are increasingly being conducted in both low and middle income countries partly because the cost of research and development is cheaper[30]. And while volunteers’ reasons for participating or not may change over time, we feel that these results showing a significant disconnect between willingness and actual participation should still serve to remind investigators to be cautious when interpreting hypothetical willingness-to-participate surveys.

This study from Kenya will form basis for reference that can be used to improve recruitment in clinical trials.

### Conclusion

Reported willingness to join a clinical trial does not always translate into actual enrolment. Researchers planning to use “banked” participants for a clinical vaccine trial will likely need to apply multiple supplemental approaches to adequately recruit participants such as: direct recruitment from the community, passive approach, and snowballing. While our example of lower-risk volunteers suitable for phase I clinical trials may not necessarily apply to larger HIV prevention trials, future studies on willingness to participate in future HIV vaccine clinical trials ought to include detailed information regarding the clinical trial requirements to allow participants make an informed decision on willingness.

## Supporting information

S1 Appendix Observational study questionnaire. (PDF)
