# Supplementary material for: Reported willingness to participate in a hypothetical HIV vaccine trial and its translation to actual participation among healthy adults—Experience from Kenya
Source: PLoS One. 2018 Nov 2;13(11):e0206656. doi: 10.1371/journal.pone.0206656 (PMC6214541; doi:10.1371/journal.pone.0206656)
Supplement: S1 Appendix — (PDF) [file pone.0206656.s001.pdf]

SURVEY QUESTIONS

Health:

1. How would you describe your present state of health?
2. Have you ever had any allergies to medicines or foods?
3. Are you currently taking any medicine prescribed by a doctor, or self-prescribed?
4. If yes, what medication are you taking and what is for?

Life-style:

1. How would you describe your sexual orientation?
2. Are you sexually active at the present time?
3. Are you abstaining from sexual intercourse?
4. Do you have any cause to believe that you might be infected with HIV?
5. Have you ever used self-injected drugs?
6. How many sexual partners have you had in the last year? Total number regular sexual partners in the last year, Total number casual sexual partners in the last year .
7. Do you partner(s) use condoms with your regular partner(s)?
8. Do you use condoms with your casual partner(s)?
9. Has/have any of your partner(s) ever self-injected drugs? When was the latest occasion of unprotected sexual activity with this partner?
10. Have you been diagnosed with/treated for a sexually transmitted infection in the last 6 months?
11. Have you ever had an HIV test performed on you?
12. If yes please provide the dates for the HIV test(s) What was the test(s) result(s)
13. Has/have any of your sexual partner(s) tested positive for HIV?

Family and Contraception

1. Do you have any children?
2. Are you/is your partner(s) using contraception?
3. If yes are you/your partner(s) using

Volunteering for this study and a future HIV vaccine clinical trial:

1. How did you first learn about KAVI
2. What motivated you to volunteer for this study (give all your possible reasons)?
3. Have you told anybody about you volunteering? If Yes, why; if No why?
4. If yes whom did you tell?
5. Did anybody object to you volunteering for this study?
6. If yes who objected?
7. Why did this person object to you volunteering
8. Have you ever participated in a clinical trial before?
9. If you were invited to receive information and consent to participate in a clinical trial/study, would you be interested and willing to volunteer?

   Please give reasons for your answer.
10. If the clinical trial involves testing an HIV vaccine, would you be interested in participating?

    Please give reasons for your answer.
11. Is there anything you would like to ask or to be clarified?
